# Cigarette smoking habits and attitudes among rheumatoid arthritis patients at a tertiary centre in South Africa

**DOI:** 10.4102/jcmsa.v4i1.350

**Published:** 2026-03-19

**Authors:** Simon J. le Roux, Herman Bagula, Richard van Zyl-Smit, Bridget Hodkinson

**Affiliations:** 1 Department of Medicine, Faculty of Health Sciences, University of Cape Town, Cape Town, South Africa

**Keywords:** rheumatoid arthritis, smoking, nicotine dependence, comorbidities, South Africa

## Abstract

**Background:**

People living with rheumatoid arthritis (RA) who smoke cigarettes are known to have worse outcomes with regard to disease control, extra-articular complications and comorbidities. Data regarding this from sub-Saharan Africa is lacking. This study aims to describe the prevalence of cigarette smoking and explore disease control, comorbidities and attitudes of smoking among RA patients in an outpatient clinic at a tertiary hospital.

**Methods:**

A cross-sectional study of consenting adult outpatients with RA was conducted. Demographic, clinical and patient-reported outcome measures, together with a questionnaire about smoking and Fagerström test for nicotine dependence, were collated.

**Results:**

Of 632 patients (536 female participants), the mean (standard deviation) age and disease duration were 55.4 (13.0) and 10.1 (9.3) years, and 74.1% had two or more comorbidities. Of 218 (34.5%) smokers, more men smoked (*p* = 0.0002). Compared to non-smokers, smokers had lower body mass index (*p* = 0.01), higher incidence of chronic obstructive pulmonary disease (*p* < 0.005) and rheumatoid factor positivity (*p* = 0.006), and higher anxiety scores (*p* = 0.048) with more impairment in usual activities (*p* = 0.05). No significant differences in disease activity, extra-articular disease, or in disability, fatigue, depression, or pain scores were observed. The most common reasons for smoking were emotional support (45.8%), nicotine craving (30.5%) and pain control (25.2%). The Fagerström score revealed mild, moderate and severe nicotine dependence in 67.5%, 24.4%, and 7.5%, respectively.

**Conclusion:**

One in three patients with RA actively smoked. Those who smoked had more pain, anxiety, and depression but with low nicotine dependency scores.

**Contribution:**

The approach to tobacco cessation should occur in parallel with optimal pain, behavioural support and disease control.

## Introduction

Cigarette smoking is recognised as a major risk factor for the development of rheumatoid factor (RF) and the subsequent development of rheumatoid arthritis (RA). In one subset grouping of RA patients with anti-citrullinated antibody-positive arthritis, a combination of the environmental risk factor of smoking and a genetic susceptibility of Human Leukocyte Antigen – DR isotype (HLA – DR) shared epitope showed a significantly increased relative risk for developing RA. Among cigarette smokers, persons with a single copy of the risk gene showed a relative risk of 6.5 and those with double copies a relative risk of 21.0.^1,2^

Once RA is established, cigarette smokers have increased joint inflammation with higher swollen joint counts and elevated inflammatory markers, radiological progression, a higher incidence of extra-articular manifestations, including nodules and interstitial lung disease, together with comorbidities such as an increased rate of infections, cardiovascular events and osteoporosis.^3^ Active smoking can result in a reduced response to treatment, in particular methotrexate and tumour necrosis factor inhibitors, with variable outcomes in current smokers on disease control, joint destruction, inflammatory markers and pain control.^3,4,5,6^

In South Africa (SA), the prevalence of smoking has shown a steady decline over the years as a result of many governmental health intervention strategies. This is similar to the trend in other low- to middle-income countries.^7^ The highest prevalence of smoking in SA is in the Western Cape and Northern Cape, and this has contributed to a high burden of both communicable and non-communicable diseases.^8,9^

The prevalence of smoking among RA patients in SA, including knowledge about smoking and joint disease and patient attitudes to quitting, is unknown. In light of the multifaceted negative impact smoking has on RA patients, documenting and treating smoking habits and nicotine dependence together with comorbidities in this population plays an important role in long-term disease management.^10^ We undertook this cross-sectional study of RA patients attending an academic state-sector hospital with the aim of gaining insight into the prevalence of cigarette smoking, as well as improving our understanding of patient beliefs and readiness to quit. Our primary objective was to describe the prevalence of smoking among RA patients in an outpatient clinic at a tertiary-level public hospital in SA. Our secondary objectives were to: (1) document RA disease control, comorbidities and extra-articular disease in these patients compared to non-smokers or ex-smokers; (2) explore RA patients’ knowledge about smoking and RA disease; and (3) measure nicotine dependence and attitudes to smoking and quitting. Provisional results of this research have been published as a congress abstract.^11^

## Research methods and design

### Study design

This was a cross-sectional study recruiting patients with a diagnosis of RA attending the arthritis outpatient clinics at a tertiary academic hospital in the Western Cape, SA. Over an 18-month period (February 2021–August 2022), consecutive adults (≥ 18 years) meeting the EULAR/ACR 2010 RA Classification Criteria^12^ were invited to participate.

### Data collection

Clinico-demographic particulars, including age, biological sex, features of RA (seropositivity, duration of illness, therapy and current disease activity as measured by the Clinical Disease Activity Index [CDAI]), were captured along with comorbid conditions and anthropometric details. Patient-reported outcome measures (PROMs) endorsed by the 2010 Outcome Measures in Rheumatology Meeting were recorded.^13,14^ These included the EuroQol (EQ-5D), Health Assessment Questionnaire-Disability Index (HAQ-DI), Hospital Anxiety and Depression Scale (HADS), Brief Pain Inventory (BPI) and functional assessment of chronic illness therapy (FACIT) Fatigue Score.^15,16,17,18,19,20^ A poor socio-economic setting (SES) was defined using a pooled index, if any of the following were present: the highest level of education as primary school or less; more than seven persons living in the household; washing clothes by hand (i.e. no access to a domestic washing machine).^21^

Participants completed a smoking-related questionnaire detailing smoking history (past or current), age of initiation, pack-year history, current cigarette consumption, attitude to quitting and previous quitting attempts. Patients were also asked if they felt that smoking worsened their RA, and if their arthritis doctors had advised quitting. Patients’ reasons for smoking and their impressions of the benefits of quitting were explored through multiple-choice questions in which one or more answer options could be selected, with space for comments or details. Participants who identified as current smokers completed the Fagerström test to assess their level of nicotine dependence, and were asked about their interest in smoking cessation, with an offer for referral to the hospital smoking cessation clinic.^22^

### Statistical analysis

Numerical variables were presented as mean and standard deviation, and categorical variables were reported as frequencies and proportions. The *T*-test and chi-squared were used to compare smokers to non-smokers, and Spearman’s rank correlation tests were applied. Multivariable linear regression analyses were conducted to assess the independent association between smoking and variables found to be significant on univariate analysis. Stata 10 software (StataCorp, US) and Microsoft Excel were used. A *p*-value of less than 0.05 was considered significant.

### Ethical considerations

Ethical clearance to conduct this study was obtained from the University of Cape Town Faculty of Health Sciences Human Research Ethics Committee (No. HREC REF 179/2021). All participants signed informed consent prior to their inclusion in the study.

## Results

Of 632 participants (536 were female), the mean (standard deviation [s.d.]) age and disease duration were 55.4 (13.0) and 10.1 (9.3) years, respectively (Appendix 1 Table 1-A1). Over a third (*n* = 218, 34.5%) self-identified as current smokers, with 89 (14.1%) ex-smokers. A poor SES was noted in 64.0% of participants, and the group overall had poor anxiety, depression, fatigue, pain severity and pain interference scores, with moderate to severe functional disability. *Most patients exhibited low disease activity* (48.9%), but the mean patient global score was moderate. Comparing smokers to non-smokers, smokers were more likely to be male and have a significantly lower body mass index. Smokers were more likely to be RF positive and were more likely to have rheumatoid nodules, although this was not statistically significant. There were no differences in age of onset, level of education and family history.

Comparing the disease activity and PROMs of smokers and non-smokers, smokers showed significantly worse anxiety scores, and more impairment on the EQ-5D domains of usual activities, anxiety and depression, with no statistical difference between the two groups for CDAI score and its components, complications of RA, HAQ-DI or HADS depression scores. Smokers were significantly more likely to have chronic obstructive pulmonary disease (COPD) than non-smokers, while other comorbidities did not have significant difference. The main comorbidities were hypertension (58.9% of all patients), diabetes mellitus (27.9%), dyslipidaemia (25.3%) and obesity (7%). Multivariable analyses revealed that smoking was significantly associated only with functional disability as measured by the HAQ-DI (β = –0.26, 95% CI [confidence interval] – 0.48 to –0.05, *p* = 0.018).

More than half of the patients (56.1%) reported starting smoking at an age of 18 years or younger. The total number of cigarettes smoked per day was reported as less than 10 in 72.8% of individuals. The major reasons participants identified for ongoing smoking were emotional support (45.8%), nicotine craving (30.5%) and pain control (25.2%). Less commonly listed reasons included social factors (22.9%), boredom (20.0%), and coping strategies (17.6%). Many (50.6%) reported that living with RA makes it difficult to quit smoking. Only 48.0% of patients were aware that smoking worsened arthritis, yet 74.4% reported receiving advice to quit smoking from their arthritis doctors. Patients who claimed they smoked for pain control had higher pain severity scores than those who did not feel pain control was a reason they smoked (5.9 (1.6) vs 4.7 (2.0), *p* < 0.001). Those smoking for emotional support had higher anxiety scores (10.6 (4.9) vs 7.9 (4.5), *p* < 0.001).

When asked to list the benefits of smoking cessation, the most common responses were: lower risks of heart attack (74.8%), cancer (59.5%) and emphysema (54.0%). Half of the smokers felt that quitting would lead to better control of arthritis (49.7%); others listed financial savings (47.9%), better fitness (37.4%), no longer smelling of smoke (32.5%) and approval from partner or family members (31.2%) as advantages of quitting. Almost half (45.6%) had previously quit for more than 3 months. A small percentage (16.8%) reported being ‘desperate to quit’, and the majority were ambivalent or not interested: ‘maybe give it a try’ (44.2%), ‘not right now’ (32.6%).

The majority of current smokers (67.5%) had mild nicotine dependence as per the Fagerström test, a quarter (24.4%) moderate and only 7.5% severe dependence. The Fagerström score positively correlated with both depression and anxiety scores: *r* 0.28 (*p* < 0.005) and *r* 0.20 (*p* = 0.02) respectively.

## Discussion

In this cross-sectional study of RA patients attending a tertiary referral centre, over a third of participants were smokers, with a further 14.1% previous smokers. This prevalence of smoking is high in comparison with the national prevalence of 17.6%, but similar to regional numbers, where the smoking prevalence in the Western Cape is 32.9%.^8^ Elsewhere, the prevalence among RA patients shows wide regional variability, ranging from 0.9% (Morocco) to 48% (Austria).^23,24,25^

In our study, we found that more male participants smoked, in keeping with national demographic trends, and smokers had a significantly lower body mass index (BMI) with a higher prevalence of COPD.^8,26^ Seropositivity, nodules and anxiety were more prevalent in smokers, which has been reported elsewhere.^27,28^ We found no significant difference among the PROMs. Many participants in our study reported emotional support and pain control as primary reasons for continuing their smoking habit. These subgroups had higher pain severity and anxiety scores, respectively, reflecting the challenges of living with RA, pain and anxiety and perhaps demonstrating that cigarette smoking is felt to ameliorate these problems.

The association between smoking, pain, anxiety and depression has been well described.^5,29,30^ Current tobacco smoking worsens pain, and early stages of nicotine withdrawal amplify pain. Smoking has been associated with other chronic pain syndromes, with worse outcomes reported in pain treatment programmes. Anxiety and depression are more prevalent among smokers relative to non-smokers and in patients experiencing chronic pain. A complex bi-directional relationship ensues with pain and smoking associated with or worsening anxiety and depression, and the negative effects of poor mental health on pain, pain management, smoking habits, and smoking cessation. Improved control of RA disease, better nonpharmacological and pharmacological pain management, together with attention to mental health care, may improve PROMs in conjunction with smoking cessation programmes.^31,32^

Smoking is a modifiable cardiovascular risk factor.^33^ The comorbidities of non-communicable and lifestyle diseases are prevalent in our RA patients, with the vast majority having two or more comorbidities, including hypertension, dyslipidaemia, diabetes and obesity. Each of these individually contributes towards a significant cardiovascular risk profile in RA patients.^34,35^ Smokers have lower BMIs due to appetite suppression, metabolic effects on lipid and glucose pathways and sympathetic activation. However, this does not offset the cardiovascular risks of smoking.^36,37^

Of concern is that less than half of the participants believed that smoking worsened their arthritis, although most reported having received smoking cessation advice from their arthritis clinic team. This is an area for improvement and should be ongoing at every clinic visit. Encouragingly, a minority of patients have severe nicotine dependence, with most patients having mild dependence. Similarly, low levels of nicotine dependence but high levels of depression have been reported at our local smoking cessation clinic.^38^ This, along with the long time to return to a non-smoker risk level, highlights the importance of early and repeated interventions for smoking cessation.^39^

The high cost of pharmacological therapies available for smoking cessation in low to middle-income countries limits their availability. The majority of smokers in the current study are not interested in or ambivalent about smoking cessation, despite low nicotine dependence. Simple and affordable strategies, including reading material, education, and brief interventions are likely to increase the interest in quitting, with readily available support for those who are actively interested in quitting. Addressing the underlying level of disease control and associated anxiety and depression in parallel with smoking cessation advice is likely to yield the best results, given the high prevalence of smoking.

Several limitations of this study can be highlighted. This is a single-centre study in the Western Cape with high smoking prevalence, which may limit its generalisability to other RA centres. As participation was voluntary, smoking may be under-reported. Smoking status and pack-year history were self-reported; thus, the true prevalence may be under-reported. Other forms of nicotine delivery, for example, chewing tobacco, vaping, or the use of cannabis or other recreational drugs, were not evaluated in this research project, and have been shown to be prevalent.^40^

## Conclusion

The prevalence of smoking among RA patients in the Western Cape is high and strongly associated with pain, anxiety and depression. Many smokers with RA were unaware of the direct negative effects of smoking on their joint disease, despite having knowledge of the risk of cardiovascular, cancer and respiratory disease. It is critical to interrupt the cycle of uncontrolled disease with further smoking-induced inflammation. A multidisciplinary approach addressing mental health, joint pain and reduced quality of life along with smoking-specific behavioural modification and nicotine replacement therapy, where needed, is required.

## Appendix 1

**TABLE 1-A1:** The characteristics of participants with rheumatoid arthritis.

Variable	Sub-variable	All patients (*n* = 632)				Smokers (*n* = 218)				Non-smokers (*n* = 414)				*p*-value
		*n*	%	Mean	s.d.	*n*	%	Mean	s.d.	*n*	%	Mean	s.d.	
Demographic details	Males	96	15.2	-	-	49	22.5	-	-	47	11.4	-	-	0.0002
	Age enrolment (years)	-	-	55.4	13.0	-	-	54.1	11.6	-	-	56.1	13.7	ns
	Disease duration (years)	-	-	10.1	9.3	-	-	9.4	8.5	-	-	10.4	9.7	ns
	BMI (Kg/m^2^)	-	-	31.5	8.1	-	-	29.7	7.4	-	-	32.9	9.6	< 0.001
	Poor SES	405	64.0	-	-	146	67	-	-	259	62.6	-	-	ns
Comorbidities	2 or more comorbidities	468	74.1	-	-	154	70.6	-	-	314	75.8	-	-	ns
	Hypertension	372	58.9	-	-	116	53.2	-	-	256	61.8	-	-	0.04
	Diabetes mellitus	176	27.9	-	-	53	24.3	-	-	123	29.7	-	-	ns
	Dyslipidaemia	160	25.3	-	-	49	22.5	-	-	111	26.8	-	-	ns
	Obesity	44	7	-	-	12	5.5	-	-	32	7.7	-	-	ns
	COPD	29	4.6	-	-	18	8.3	-	-	11	2.7	-	-	0.002
Disease particulars	CDAI	-	-	14.3	11.8	-	-	14.3	12.6	-	-	14.2	11.4	ns
	• Tender Joint Count	-	-	3.2	4.5	-	-	3.1	4.6	-	-	3.2	4.5	ns
	• Swollen Joint Count	-	-	3.1	4.4	-	-	3.3	5.0	-	-	2.9	4.1	ns
	• Physician Global Assessment	-	-	3.3	2.7	-	-	3.2	2.7	-	-	3.35	2.76	ns
	• Patient Global Assessment	-	-	4.8	2.7	-	-	4.9	2.8	-	-	4.79	2.7	ns
	CDAI low activity	309	48.9	-	-	111	50.9	-	-	198	47.8	-	-	ns
	CDAI moderate activity	185	29.3	-	-	62	28.4	-	-	123	29.7	-	-	ns
	CDAI high activity	134	21.2	-	-	44	20.9	-	-	90	21.7	-	-	ns
	RF positive	464	73.4	-	-	175	80.3	-	-	289	69.8	-	-	0.006
	Nodules	144	22.8	-	-	59	27	-	-	85	20.5	-	-	ns
PROM	Total HAQ-DI	-	-	1.5	0.7	-	-	1.5	0.7	-	-	1.5	0.7	ns
	Pain severity score	-	-	4.8	2.0	-	-	4.9	2.0	-	-	4.8	2.0	ns
	Pain interference score	-	-	4.4	2.4	-	-	4.4	2.5	-	-	4.4	2.4	ns
	Anxiety score	-	-	8.3	4.8	-	-	8.8	4.7	-	-	8.0	4.8	0.049
	Depression score	-	-	9.1	6.1	-	-	9.5	6.3	-	-	8.9	4.8	ns
	FACIT score	-	-	28.3	13.3	-	-	27.5	12.9	-	-	28.8	13.4	ns
	EQ-5D level sum score	-	-	9.1	2.2	-	-	9.2	2.5	-	-	9.1	2.0	ns
	• Mobility	-	-	1.7	0.5	-	-	1.7	0.5	-	-	1.7	0.5	ns
	• Self care	-	-	1.8	0.7	-	-	1.8	0.6	-	-	1.75	0.6	ns
	• Usual activities	-	-	1.8	0.5	-	-	1.9	0.5	-	-	1.77	0.5	0.05
	• Pain and discomfort	-	-	2.4	0.6	-	-	2.4	0.6	-	-	2.4	0.6	ns
	• Anxiety and depression	-	-	1.7	0.6	-	-	1.8	0.6	-	-	1.7	0.6	0.04

BMI, body mass index; SES, socio-economic status; COPD, chronic obstructive pulmonary disease; HAQ-DI, health assessment questionnaire disability index; FACIT, functional assessment of chronic illness therapy; CDAI, clinical disease activity index; RF, rheumatoid factor; ns, not significant; PROM, patient-reported outcome measures; s.d., standard deviation; EQ-5D, EuroQol-5 dimensions.

## References

[1] Sugiyama D, Nishimura K, Tamaki K, et al. Impact of smoking as a risk factor for developing rheumatoid arthritis: A meta-analysis of observational studies. Ann Rheum Dis. 2010;69(1):70–81. 10.1136/ard.2008.096487 19174392

[2] Klareskog L, Stolt P, Lundberg K, et al. A new model for an etiology of rheumatoid arthritis: Smoking may trigger HLA-DR (shared epitope)-restricted immune reactions to autoantigens modified by citrullination. Arthritis Rheum. 2006;54(1):38–46. 10.1002/art.21575 16385494

[3] Vittecoq O, Richard L, Banse C, Lequerre T. The impact of smoking on rheumatoid arthritis outcomes. Joint Bone Spine. 2018;85(2):135–138. 10.1016/j.jbspin.2017.12.004 29246527

[4] Westhoff G, Rau R, Zink A. Rheumatoid arthritis patients who smoke have a higher need for DMARDs and feel worse, but they do not have more joint damage than non-smokers of the same serological group. Rheumatology. 2008;47(6):849–854. 10.1093/rheumatology/ken057 18390589

[5] Zale EL, Maisto SA, Ditre JW. Anxiety and depression in bidirectional relations between pain and smoking: Implications for smoking cessation. Behav Modif. 2016;40(1–2):7–28. 10.1177/0145445515610744 26467214 PMC4834030

[6] Nyhall-Wahlin BM, Petersson IF, Nilsson JA, Jacobsson LT, Turesson C, BARFOT Study Group. High disease activity disability burden and smoking predict severe extra-articular manifestations in early rheumatoid arthritis. Rheumatology (Oxford). 2009;48(4):416–420. 10.1093/rheumatology/kep004 19213849

[7] World Health Organization. WHO report on the global tobacco epidemic 2021: Addressing new and emerging products. Geneva: World Health Organization; 2021.

[8] Reddy P, Zuma K, Shisana O, Jonas K, Sewpaul R. Prevalence of tobacco use among adults in South Africa: Results from the first South African National Health and Nutrition Examination Survey. S Afr Med J. 2015;105(8):648–655. 10.7196/SAMJnew.7932 26449697

[9] Gouda HN, Charlson F, Sorsdahl K, et al. Burden of non-communicable diseases in sub-Saharan Africa, 1990–2017: Results from the Global Burden of Disease Study 2017. Lancet Glob Health. 2019;7(10):e1375–e1387. 10.1016/S2214-109X(19)30374-2 31537368

[10] Solomon A, Stanwix AE, Castañeda S, et al. Points to consider in cardiovascular disease risk management among patients with rheumatoid arthritis living in South Africa, an unequal middle income country. BMC Rheumatol. 2020;4(1):1–16. 10.1186/s41927-020-00139-2 32550295 PMC7296622

[11] Le Roux S, Bagula H, Didi SA, Van Zyl-Smit R, Hodkinson B. AB0364 cigarette smoking amongst rheumatoid arthritis patients in a Tertiary Center in South Africa. Ann Rheum Dis. 2023;82(Suppl 1):1366. 10.1136/annrheumdis-2023-eular.4039

[12] Aletaha D, Neogi T, Silman AJ, et al. 2010 rheumatoid arthritis classification criteria: An American College of Rheumatology/European League Against Rheumatism collaborative initiative. Arthritis Rheum. 2010;62(9):2569–2581. 10.1002/art.27584 20872595

[13] Aletaha D, Smolen J. The Simplified Disease Activity Index (SDAI) and the Clinical Disease Activity Index (CDAI): A review of their usefulness and validity in rheumatoid arthritis. Clin Exp Rheumatol. 2005;23(5):S100. 10.1016/j.berh.2007.02.004 16273793

[14] Petkovic J, Epstein J, Buchbinder R, et al. Toward ensuring health equity: Readability and cultural equivalence of OMERACT patient-reported outcome measures. J Rheumatol. 2015;42(12):2448–2459. 10.3899/jrheum.141168 26077410 PMC4765994

[15] Hawker GA, Mian S, Kendzerska T, French M. Measures of adult pain: Visual Analog Scale for Pain (VAS Pain), Numeric Rating Scale for Pain (NRS Pain), McGill Pain Questionnaire (MPQ), Short-Form McGill Pain Questionnaire (SF-MPQ), Chronic Pain Grade Scale (CPGS), Short Form-36 Bodily Pain Scale (SF-36 BPS), and Measure of Intermittent and Constant Osteoarthritis Pain (ICOAP). Arthritis Care Res (Hoboken). 2011;63(Suppl 11):S240–S252. 10.1002/acr.20543 22588748

[16] Hurst NP, Kind P, Ruta D, Hunter M, Stubbings A. Measuring health-related quality of life in rheumatoid arthritis: Validity, responsiveness and reliability of EuroQol (EQ-5D). Br J Rheumatol. 1997;36(5):551–559. 10.1093/rheumatology/36.5.551 9189057

[17] Kirwan JR, Reeback JS. Stanford Health Assessment Questionnaire modified to assess disability in British patients with rheumatoid arthritis. Br J Rheumatol. 1986;25(2):206–209. 10.1093/rheumatology/25.2.206 3708236

[18] Bjelland I, Dahl AA, Haug TT, Neckelmann D. The validity of the Hospital Anxiety and Depression Scale. An updated literature review. J Psychosom Res. 2002;52(2):69–77. 10.1016/s0022-3999(01)00296-3 11832252

[19] Cella D, Yount S, Sorensen M, Chartash E, Sengupta N, Grober J. Validation of the Functional Assessment of Chronic Illness Therapy Fatigue Scale relative to other instrumentation in patients with rheumatoid arthritis. J Rheumatol [serial online]. 2005 [cited 2021 Mar 21];32(5):811–819. Available from: https://www.ncbi.nlm.nih.gov/pubmed/15868614 15868614

[20] Tan G, Jensen MP, Thornby JI, Shanti BF. Validation of the Brief Pain Inventory for chronic nonmalignant pain. J Pain. 2004;5(2):133–137. 10.1016/j.jpain.2003.12.005 15042521

[21] Calixto OJ, Anaya JM. Socioeconomic status. The relationship with health and autoimmune diseases. Autoimmun Rev. 2014;13(6):641–654. 10.1016/j.autrev.2013.12.002 24418307

[22] Heatherton TF, Kozlowski LT, Frecker RC, Fagerstrom KO. The Fagerstrom Test for Nicotine Dependence: A revision of the Fagerstrom Tolerance Questionnaire. Br J Addict. 1991;86(9):1119–1127. 10.1111/j.1360-0443.1991.tb01879.x 1932883

[23] Ye D, Mao Y, Xu Y, Xu X, Xie Z, Wen C. Lifestyle factors associated with incidence of rheumatoid arthritis in US adults: Analysis of National Health and Nutrition Examination Survey database and meta-analysis. BMJ Open. 2021;11(1):e038137. 10.1136/bmjopen-2020-038137 PMC7843328 33500279

[24] Dougados M, Soubrier M, Antunez A, et al. Prevalence of comorbidities in rheumatoid arthritis and evaluation of their monitoring: Results of an international, cross-sectional study (COMORA). Ann Rheum Dis. 2014;73(1):62–68. 10.1136/annrheumdis-2013-204223 24095940 PMC3888623

[25] Ranganath VK, Maranian P, Elashoff DA, et al. Comorbidities are associated with poorer outcomes in community patients with rheumatoid arthritis. Rheumatology (Oxford). 2013;52(10):1809–1817. 10.1093/rheumatology/ket224 23813577 PMC3775293

[26] Brunet L, Pai M, Davids V, et al. High prevalence of smoking among patients with suspected tuberculosis in South Africa. Eur Respir J. 2011;38(1):139–146. 10.1183/09031936.00137710 21148230 PMC5454478

[27] Watad A, Bragazzi NL, Adawi M, et al. Anxiety disorder among rheumatoid arthritis patients: Insights from real-life data. J Affect Disord. 2017;213:30–34. 10.1016/j.jad.2017.02.007 28188994

[28] Ishikawa Y, Ikari K, Hashimoto M, et al. Shared epitope defines distinct associations of cigarette smoking with levels of anticitrullinated protein antibody and rheumatoid factor. Ann Rheum Dis. 2019;78(11):1480–1487. 10.1136/annrheumdis-2019-215463 31427439

[29] Ditre JW, Brandon TH, Zale EL, Meagher MM. Pain, nicotine, and smoking: Research findings and mechanistic considerations. Psychol Bull. 2011;137(6): 1065–1093. 10.1037/a0025544 21967450 PMC3202023

[30] Hooten MW, Shi Y, Gazelka HM, Warner DO. The effects of depression and smoking on pain severity and opioid use in patients with chronic pain. Pain. 2011;152(1):223–229. 10.1016/j.pain.2010.10.045 21126821 PMC3026611

[31] Lu B, Rho YH, Cui J, et al. Associations of smoking and alcohol consumption with disease activity and functional status in rheumatoid arthritis. J Rheumatol. 2014;41(1):24–30. 10.3899/jrheum.130074 24293566 PMC4017580

[32] Gwinnutt JM, Wieczorek M, Balanescu A, et al. 2021 EULAR recommendations regarding lifestyle behaviours and work participation to prevent progression of rheumatic and musculoskeletal diseases. Ann Rheum Dis. 2023;82(1):48–56. 10.1136/annrheumdis-2021-222020 35260387

[33] Roelsgaard IK, Ikdahl E, Rollefstad S, et al. Smoking cessation is associated with lower disease activity and predicts cardiovascular risk reduction in rheumatoid arthritis patients. Rheumatology (Oxford). 2020;59(8):1997–2004. 10.1093/rheumatology/kez557 31782789 PMC7382591

[34] Dessein PH, Solomon A, Hollan I. Metabolic abnormalities in patients with inflammatory rheumatic diseases. Best Pract Res Clin Rheumatol. 2016;30(5):901–915. 10.1016/j.berh.2016.10.001 27964795

[35] Nurmohamed MT, Heslinga M, Kitas GD. Cardiovascular comorbidity in rheumatic diseases. Nat Rev Rheumatol. 2015;11(12):693–704. 10.1038/nrrheum.2015.112 26282082

[36] Harris KK, Zopey M, Friedman TC. Metabolic effects of smoking cessation. Nat Rev Endocrinol. 2016;12(5):299–308. 10.1038/nrendo.2016.32 26939981 PMC5021526

[37] Visseren FLJ, Mach F, Smulders YM, et al. 2021 ESC guidelines on cardiovascular disease prevention in clinical practice. Eur Heart J. 2021;42(34):3227–3337. 10.1093/eurheartj/ehab484 34458905

[38] Tadzimirwa GY, Day C, Esmail A, et al. Challenges for dedicated smoking cessation services in developing countries. S Afr Med J. 2019;109(6):431–436. 10.7196/SAMJ.2019.v109i6.13631 31266563

[39] Lahiri M, Morgan C, Symmons DP, Bruce IN. Modifiable risk factors for RA: Prevention, better than cure? Rheumatology (Oxford). 2012;51(3):499–512. 10.1093/rheumatology/ker299 22120459 PMC3281496

[40] Govind N, Ally MM, Tikly M, Anderson R, Hodkinson B, Meyer PW. Pitfalls in the assessment of smoking status detected in a cohort of South African RA patients. Rheumatol Int. 2016;36(10):1365–1369. 10.1007/s00296-016-3527-y 27393331

